# Terretonin as a New Protective Agent against Sepsis-Induced Acute Lung Injury: Impact on SIRT1/Nrf2/NF-κBp65/NLRP3 Signaling

**DOI:** 10.3390/biology10111219

**Published:** 2021-11-22

**Authors:** Gamal A. Mohamed, Sabrin R. M. Ibrahim, Dina S. El-Agamy, Wael M. Elsaed, Alaa Sirwi, Hani Z. Asfour, Abdulrahman E. Koshak, Sameh S. Elhady

**Affiliations:** 1 Department of Natural Products and Alternative Medicine, Faculty of Pharmacy, King Abdulaziz University, Jeddah 21589, Saudi Arabia; asirwi@kau.edu.sa (A.S.); aekoshak@kau.edu.sa (A.E.K.); ssahmed@kau.edu.sa (S.S.E.); 2 Preparatory Year Program, Batterjee Medical College, Jeddah 21442, Saudi Arabia; sabrin.ibrahim@bmc.edu.sa; 3 Department of Pharmacognosy, Faculty of Pharmacy, Assiut University, Assiut 71526, Egypt; 4 Department of Pharmacology and Toxicology, Faculty of Pharmacy, Mansoura University, Mansoura 35516, Egypt; dinaagamy@mans.edu.eg; 5 Department of Pharmacology and Toxicology, College of Pharmacy, Taibah University, Medina 30078, Saudi Arabia; 6 Department of Anatomy and Embryology, Faculty of Medicine, Mansoura University, Mansoura 35516, Egypt; wzaarina@mans.edu.eg; 7 Department of Medical Microbiology and Parasitology, Faculty of Medicine, King Abdulaziz University, Jeddah 21589, Saudi Arabia; hasfour@kau.edu.sa

**Keywords:** *Aspergillus terreus*, endophytic fungi, terretonin, anti-inflammatory, LPS, Nrf2, NF-κB

## Abstract

**Simple Summary:**

Acute lung injury (ALI) is a severe inflammatory pulmonary disorder that still causes a high mortality rate. As the current therapeutic strategies have not been effective in reducing ALI-associated mortality, searching for a novel pharmacological candidate that can suppress ALI is mandatory. In this study, we assessed the effects of terretonin (TE), a meroterpenoid against LPS (lipopolysaccharide)-induced ALI and explored the possible underlying molecular mechanisms. The results have shown the potent protective activities of TE toward LPS-induced ALI. TE enhanced the SIRT1/Nrf2 protective pathway and its associated genes. On the other hand, TE inhibited NF-κBp65/NLRP3 signaling and its downstream inflammatory mediators. Thus, this study is the first to elucidate the potential therapeutic use of TE against ALI.

**Abstract:**

Endophytic fungi are proving to be an excellent source of chemical entities with unique structures and varied bioactivities. Terretonin (TE) and its structurally related derivatives are a class of meroterpenoids, possessing the same unique tetracyclic core skeleton, which have been reported from the *Aspergillus* genus. This study was carried out to assess the potential protective effects of TE separated from the endophytic fungus *A. terreus* against LPS (lipopolysaccharide)-induced ALI (acute lung injury) in mice. The results revealed that TE alleviated pulmonary edema as it lowered both the W/D lung ratio and protein content. The inflammatory response represented by inflammatory cell infiltration into the lung tissues was greatly repressed by TE. That was supported by the improved histopathological results and also by the reduced level of myeloperoxidase in the lung. TE showed a potent antioxidant activity as it attenuated lipid peroxidative markers (malondialdehyde, 4-hydroxynonenal, and protein carbonyl) and enhanced endogenous antioxidants (reduced glutathione, superoxide dismutase, and catalase) in lung tissues. Similarly, TE increased the mRNA expression of SIRT1, Nrf2, and its genes (*HO-1*, *NQO1*, and *GCLm*). On the other hand, TE restrained the activation of NF-κB (nuclear factor-κB) in the lung. Consequently, TE depressed the pro-inflammatory cytokines: nitric oxide (NOx), TNF-α (tumor necrosis factor-α), and interleukins (IL-6 and -1β). Additionally, TE inhibited NLRP3 signaling and interrupted apoptosis by decreasing the levels of proapoptotic markers (Bax and caspase-3) and increasing the level of an anti-apoptotic marker (Bcl-2). In conclusion, TE had a remarkable protective potential on LPS-induced lung damage via antioxidant and anti-inflammatory mechanisms. This finding encourages further investigations on this promising candidate.

## 1. Introduction

Acute lung injury (ALI) is a severe ailment with an elevated morbidity rate worldwide. It is characterized by the infiltration of inflammatory PMNs (polymorphonuclear cells) into pulmonary tissue associated with disrupted vascular permeability and pulmonary edema. The exact pathology of ALI remains elusive; however, many pathogenic players such as oxidative stress, lipid peroxidation, and inflammation interact to mediate the development of ALI [1,2]. During ALI, the continuous overproduction of ROS/RNS (reactive oxygen/nitrogen species) overloads the antioxidant capacity of the tissue, causing destruction of the cellular components such as DNA, proteins, and lipids [3,4]. LPS (lipopolysaccharide) is a glycolipid obtained from the bacterial cell wall and is widely utilized to establish a rodent model of ALI [5]. LPS induces the massive generation of ROS/RNS, which causes oxidative damage to pulmonary tissue and stimulates the activation and migration of neutrophils and macrophages into the lung. These activated infiltered cells release a wide variety of pro-inflammatory cytokines such as TNF-α and ILs (interleukins), as well as profibrotic growth factors such as transforming growth factor-beta [6,7]. Simultaneously, the activation of the upstream nuclear transcriptional factor-κB (NF-κB) augments the excessive production of pro-inflammatory cytokines (e.g., ILs and TNF-α) [8,9,10]. In addition to NF-κB, the NOD-like receptor 3 (NLRP3) inflammasome contributes to the inflammatory response via induction and activation of pro-caspase-1 to release caspase-1, which subsequently induces the production of IL-1β and IL-18 from its pro-forms [9,11]. Nrf2 (nuclear factor (erythroid-derived 2)-like 2) is a substantial nuclear transcriptional factor that is activated during oxidative stress. Upon activation, Nrf2 induces the transcription of antioxidant genes (*NQO1, GCLm, GCLc*, and *HO-1*) and antioxidant enzymes (GSH and SOD), leading to the neutralization of the oxidative stress and amelioration of the oxidative injury [12,13]. SIRT1 (silent information regulator Sirtuin 1) is a NAD^+^-dependent histone deacetylase that has a pivotal role in the protection against oxidative inflammatory damage during ALI. Recently, SIRT1 has been shown to suppress pulmonary edema via alleviation of the endothelial tight junction permeability [14]. The activation of SIRT1 has been linked to the modulation of Nrf2 antioxidant signaling [15]. Furthermore, SIRT1 can counteract the inflammatory response via downregulation of the NF-κB signaling pathway [16,17]. The interplay between SIRT1, Nrf2, NF-κB, and NLRP3 pathways regulates the cellular oxidative inflammatory responses, and hence, the agents that promote the activation of SIRT1/Nrf2 and block NF-κB/NLRP3 can control the deleterious effects of LPS and attenuate ALI.

**Figure 1:**
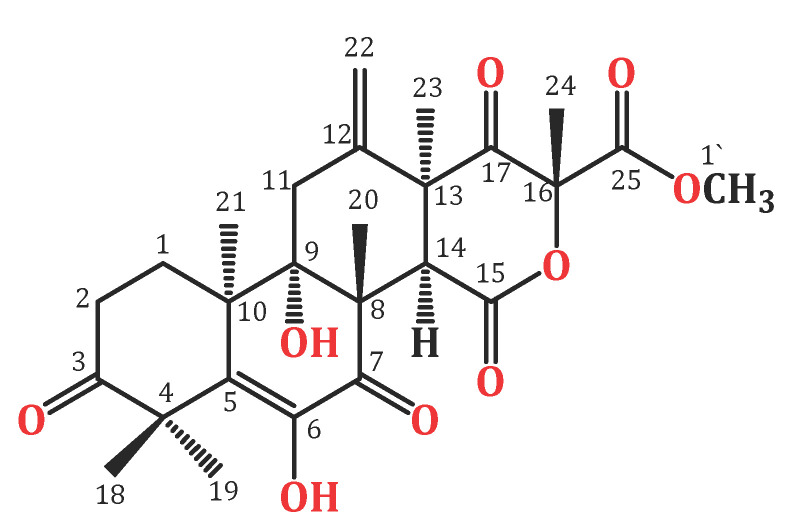
Chemical structure of terretonin (TE).

Endophytic fungi constitute a wealthy source of bioactive metabolites for agricultural, industrial, pharmaceutical, environmental, and medical applications [18,19,20,21,22,23,24]. Complex molecular architectures and structural diversity are key features of these metabolites; thus, they are considered as potential candidates for developing beneficial and novel agents [25,26,27,28,29,30,31]. Terpenes are a remarkable group of bio-metabolites, which represent the biggest class of natural metabolites [32]. Meroterpenes are a relatively small group of terpenoids reported from various natural sources such as fungi, insects, terrestrial plants, marine organisms, and lichens [33,34,35]. Terretonins, a class of the fungal meroterpenoids, have mainly been reported from the *Aspergillus* genus and possess various bioactivities [34,35,36,37,38]. During our ongoing search for exploring the bioactivities of the endophytic fungal metabolites, the protective effect of terretonin (Figure 1) isolated from *A. terreus* against sepsis-induced ALI was investigated, in addition to its molecular pathways.

## 2. Materials and Methods

### 2.1. General Procedures

ESI (electrospray ionization) MS was performed on a DECA/LCQ Thermo-finnigan mass spectrometer. A DRX850 Bruker Avance MHz was used for measuring NMR. The compound separation was achieved using a RP-18, SiO_2_, and Sephadex LH-20. TLC was performed utilizing SiO_2_60F_254_ TLC plates (0.2 mm). For detecting the compound, *p*-anisaldehyde:H_2_SO_4_ reagent and UV absorption (λ_max_ 366 and 255 nm) were employed.

### 2.2. Fungal Material

*Amberboa lipii* L. was gathered from North of Qiba, Al-Madinah Al-Munawwarah, Saudi Arabia and verified by Dr. Emad Alsherif (Faculty of Science and Arts, Khulais, Department of Biology, King Abdulaziz University). A specimen (AL-3-2020) was well-kept at the Natural Products and Alternative Medicine Department’s herbarium. *Aspergillus* *terreus* was separated from the roots’ internal tissues of *A*. *lipii*. The inner root tissues were precisely dissected under sterile conditions and placed on dextrose-potato agar plates. The fungus was specified by relying on the colonial morphological features and microscopic observation, utilizing Olympus CX31RBSF light microscopy [39], which was genetically proved by the ITS sequence analysis that was compared with related sequences at the NCBI (http://www.ncbi.nlm.nih.gov/, accessed on 25 May 2020), which exhibited a similarity of 100% with the isolate with accession number (Genbank Accession number KX694148). The fungus was kept at the Microbiology Department, Faculty of Pharmacy, King Abdulaziz University [40]. The fresh fungal culture was transported to 12 flasks (Erlenmeyer, 1 L each), containing rice solid cultures (distilled H_2_O 100 mL + 100 g commercial rice and left overnight before autoclaving). The cultures were then incubated for 30 days under septic conditions at room temperature.

### 2.3. Purification and Structural Analysis of Terretonin

**Figure 2:**
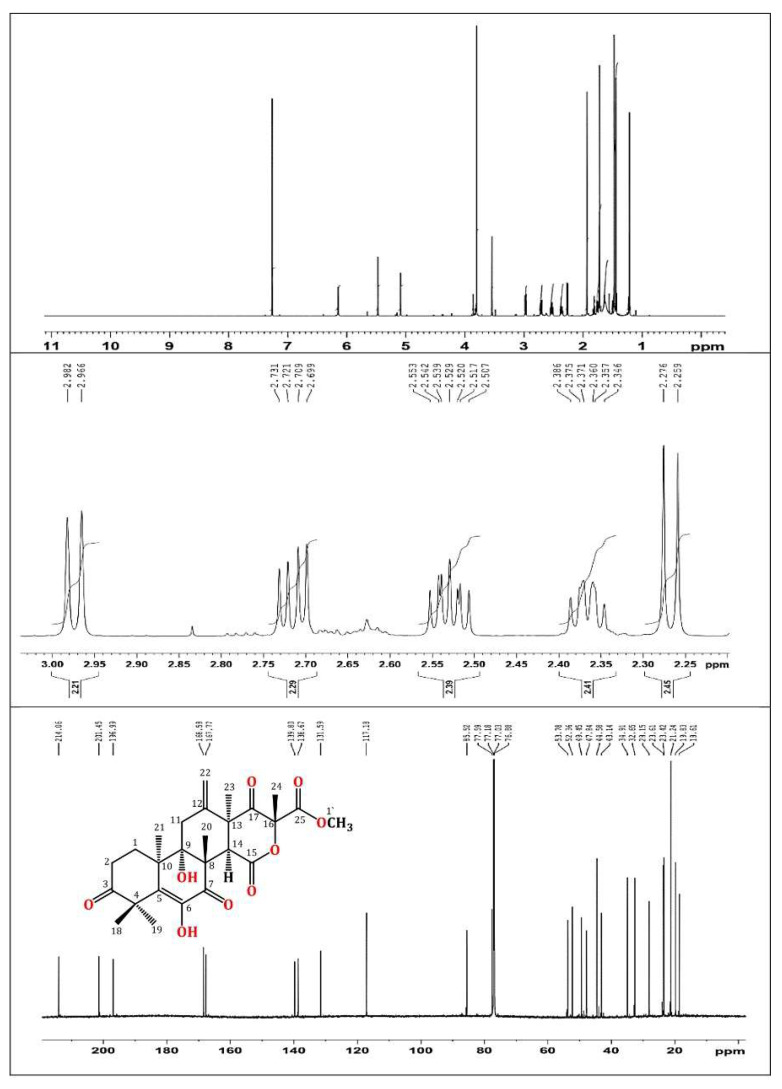
^1^H (850 MHz) and ^13^C (214 MHz) NMR spectra of terretonin in CDCl_3_.

In brief, the rice cultures of *A*. *terreus* were extracted with EtOAc. The concentrated extract was partitioned among MeOH and *n*-hexane. The total MeOH extract (12.3 g) was submitted to VLC employing *n*-hexane, CHCl_3_, and MeOH, which were individually concentrated to yield AT-I (2.1 g), AT-II (3.9 g), and AT-III (5.2 g), respectively. Fraction AT-II (3.9 g) was separated on a SiO_2_ CC utilizing an *n*-hexane:EtOAc gradient elution to afford four subfractions: AT-II-A (0.62 g), AT-II-B (1.23 g), AT-II-C (0.761 g), and AT-II-D (0.914 g). Subfraction AT-II-B (1.23 g) was subjected to a Sephadex LH-20 CC (MeOH:CHCl_3_, 90:10) to afford impure TE that was purified by an RP-18 CC (MeOH:H_2_O gradient) to give white amorphous powder, which was collected from the solution, washed with hexane, and recrystallized using the CHCl_3_:MeOH mixture at room temperature. Colorless fine needles (280 mg) were obtained from the solvent, which were identified as TE based on physical and spectral data: mp 258–259 °C; ESIMS: *m/z* 489 [M + H]^+^; IR (KBr) ν_max_ 3415, 2963, 1725, 1715, 1680, 1153 cm^−1^; ^1^H NMR (CDCl_3_, 850 MHz): δ_H_ 2.37 (H1A), 1.76 (H1B), 2.71 (H2A), 2.53 (H2B), 2.98 (H11A), 2.27 (H11B), 3.54 (H14), 1.47 (H18), 1.43 (H19), 1.93 (H20), 1.21 (H21), 5.47 (H22A), 5.09 (H22B), 1.48 (H23), 1.72 (H24), 3.80 (H1′), 6.15 (6OH); ^13^C NMR (213 MHz, CDCl_3_): δ_C_ 28.2 (C1), 32.7 (C2), 214.1 (C3), 47.8 (C4), 131.6 (C5), 138.7 (C6), 197.0 (C7), 52.4 (C8), 77.6 (C9), 43.1 (C10), 34.9 (C11), 139.8 (C12), 49.5 (C13), 44.6 (C14), 167.8 (C15), 85.5 (C16), 201.5 (C17), 21.2 (C18), 23.4 (C19), 19.8 (C20), 18.6 (C21), 117.2 (C22), 23.6 (C23), 21.2 (C24), 168.6 (C25), 53.8 (C1′) (Figure 1 and Figure 2) [34,35].

### 2.4. In Vivo Experiments

#### 2.4.1. Animals

Male Balb/c albino mice were kept under standard conditions of humidity, temperature, and 12 h dark/light cycles. They received human care during the whole experimental period and were freely allowed food and water. The study approval was obtained from the King Abdulaziz University Research Ethical Committee (no. PH-116-40) and the procedures were accomplished in strict commensurate with the rules of the Use and Care of Laboratory Animals of Saudi Arabia. 

#### 2.4.2. Experimental Design

ALI model. ALI was induced using a single intraperitoneal injection of *Escherichia coli* LPS (a dose of 10 mg/kg, IP) (O111:B4, Sigma Aldrich, St. Louis, MO, USA). Mice (25 to 30 g) were randomly separated into five groups (N = 8) and received treatment as follows: 1. Control: mice were treated with the vehicle. 2. TE: mice received TE (40 mg/kg/day, orally) for 5 days. 3. LPS: mice received LPS (10 mg/kg, i.p.). 4. TE 20 + LPS: mice received TE for 5 days (20 mg/kg/day, orally) prior to LPS treatment. 5. TE 40 + LPS: mice received TE for 5 days (40 mg/kg/day, orally) prior to LPS treatment.

After LPS injection by twenty-four hours, mice were killed by an overdose of anesthesia. After chest opening, the left lung was clamped. The right lung was perfused 3 times using sterile saline. BALF (broncho-alveolar lavage fluid) was gathered and centrifuged. BALF was stored for further analyses at −80 °C, while cell pellets were utilized for determining the cell counts. A small segment of the left lung was weighed and homogenized in phosphate buffer to obtain the supernatants. A part of the left lung was removed and washed with saline and then incubated for 24 h in formalin for histological assessment. Another part of the left lung was stored at −80 °C for further RT-PCR experiments.

#### 2.4.3. Biochemical Assessments

##### Lung W/D (Wet/Dry Weight) Ratio

A small piece of the left lung was blotted dry and weighed (W). Afterward, the piece was placed for 24 h at 80 °C in an oven and weighed (D) again. The lung W/D ratio was calculated to estimate the degree of pulmonary edema.

##### Protein Content and LDH (Lactate Dehydrogenase) Potential

These were estimated in BALF following the instructions of the available kits (Pierce-BCA Protein Assay Kit, Thermo-fisher Scientific, Waltham, MA, USA; Human, Wiesbaden, Germany, respectively).

##### Inflammatory Cell Counts

Total cell count was assessed in BALF using a hemocytometer. Cell pellets were re-suspended in saline (0.9%) and then centrifuged onto slides and stained for 8 min with Wright-Giemsa. Employing the light microscope, the differential cell counts were estimated by counting a total of 200 cells/slide at 40× magnification.

##### Myeloperoxidase (MPO) Activity

This was determined using the cell pellets remaining after lung homogenization as described previously [8].

##### Lipid Peroxidative and Antioxidant Markers

MDA (malondialdehyde), 4-HNE (4-hydroxynonenal), and protein carbonyl (PC) contents were measured in the supernatants of the lung homogenates to estimate the degree of lipid peroxidation in pulmonary tissue according to the instructions of the kits (Bio-diagnostic Co., Giza, Egypt; MyBioSource, San Diego, CA, USA). The endogenous antioxidants, GSH (reduced glutathione), CAT (catalase), SOD (superoxide dismutase), and total TAC (antioxidant capacity), were measured in the lung homogenates’ supernatant in compliance with the kits’ instructions (Bio-diagnostic Co., Giza, Egypt).

##### Nrf2 and Hemoxygenase-1 (HO-1)

The Nrf2-binding activity and level of HO-1 were assessed in the lung according to the guidelines of the kits (Active Motif, Carlsbad, CA, USA; MyBioSource, San Diego, CA, USA).

##### NF-κB and Downstream Cytokines

Levels of NF-κB, NOx (nitrite/nitrate), TNF-α, and IL-6 and -1β were estimated in the supernatants of lung homogenates employing ELISA kits (Abcam, Cambridge, MA, USA; R&D Systems, Minneapolis, MN, USA).

##### Apoptotic Markers

Levels of B-cell leukemia/lymphoma 2 (Bcl-2), Bcl-2-associated X protein (Bax), and caspase 3 (Casp-3) were determined in the supernatants of lung homogenates using ELISA kits (Cusabio Biotech CO., Shanghai, China).

#### 2.4.4. Histopathological and Immuno-Histochemical (IHC) Assessment

Fixed lung tissue was paraffinized and sectioned (4–5 µm). Specimens were stained with H&E (hematoxylin-eosin). The pulmonary injury was estimated and semiquantitatively graded as previously described [10]. IHC staining for NF-κB p65 was automatically accomplished utilizing a Ventana BenchMark XT system (Ventana Medical Systems, Oro Valley, AZ, USA) as described previously [27]. Semi-quantitative analysis was performed by utilizing open-source digital image analysis software (ImageJ, 1.46a; NIH).

#### 2.4.5. RT-PCR Assessment

Total RNA was extracted from the lung using RNeasy Mini kit (Qiagen, Germantown, MD, USA). RNA quality was checked spectrophotometrically using the A260/A280 ratio and nonpure samples were excluded. RNA was reverse-transcribed to cDNA (Quantitect-Reverse-Transcription Kit, Qiagen, Germantown, MD, USA). Quantitative RT-PCR was performed on the thermocycler Rotor-Gene Q (Qiagen, Hilden, Germany) utilizing SYBR Green (Qiagen, Germantown, MD, USA) and based on the manufacturer’s instructions. The primers’ sequences (5′–3′) used for the estimation of the targeted genes are presented in Table 1. Target mRNAs’ relative levels were normalized to β-actin and estimated utilizing the ^ΔΔ^Ct method.

#### 2.4.6. Statistical Analysis

Represented data were means ± SE (*n* = 8). The analysis was carried out utilizing one-way ANOVA and subsequently Tukey’s Kramer multiple comparisons test. *p* < 0.05 was deemed significant.

## 3. Results

There was no significant change between the control and TE 40 groups in all the measured parameters.

### 3.1. TE Lowered LPS-Induced Lung Edema and LDH Activity in BALF and Improved LPS-Induced Lung Histopathological Damage

**Figure 3:**
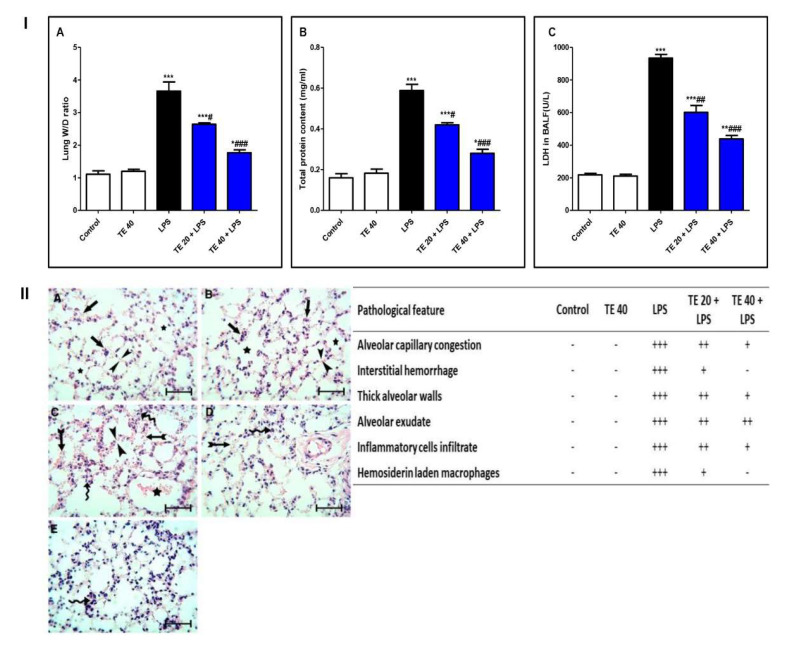
Terretonin (TE) attenuated biochemical and histopathological indices of lipopolysaccharide (LPS)-induced ALI. (**I**) (**A**) Lung W/D ratio; (**B**) total protein content; and (**C**) lactate dehydrogenase (LDH) activity in BALF. (**II**) Lung sections of: (**A**) Control and (**B**) TE 40 groups showed normal lung histology (normal alveolar capillaries (black arrows), thin alveolar walls (between arrow heads), and clear alveoli (star)). (**C**) Lungs of the LPS group exhibited many pathological features such as congested alveolar capillaries and alveolar hemorrhage (tailed black arrows), thick edematous alveolar walls (between arrow heads), edema fluid exudates in the alveoli (star), infiltration of inflammatory cells (curved arrows). (**D**) TE 20 + LPS and (**E**) TE 40 + LPS groups showed much improvement of all LPS-induced pathological changes in the lung. H&E stain ×400, scale bar 25 µm. Semiquantitative assessment of pathological changes in lung tissue in all groups. Data are the mean ± SE (*n* = 8). * *p* < 0.05, ** *p* < 0.01, *** *p* < 0.001 vs. control group; # *p* < 0.05, ## *p* < 0.01, ### *p* < 0.001 vs. LPS group (one-way ANOVA followed by Tukey’s Kramer multiple comparisons test).

LPS challenge induced remarkable pulmonary edema as there was a notable increase in the lung W/D ratio and protein content of BALF compared to normal mice (Figure 3I(A,B)). Additionally, LPS caused an elevation in LDH activity in BALF (Figure 3I(C)). However, TE pretreatment reduced the lung W/D ratio, protein content, and LDH activity compared to the LPS group.

Histopathological examination of the lung specimen of the control exhibited normal histology with no mark of any pathological change (Figure 3II). Lungs of the LPS group showed extensive injury in the form of congested alveolar capillaries accompanied by alveolar hemorrhage. There were edematous thick alveolar walls, marked inflammatory cells’ infiltration, edema fluid exudates in the alveoli, and hemosiderin-laden macrophages. On the contrary, TE + LPS-treated groups appeared protected from the injurious effects of LPS. The signs and scores of lesions were remarkably ameliorated.

### 3.2. TE Ameliorated LPS-Induced Inflammatory Cell Infiltration into the Lung and Repressed MPO Activity

**Figure 4:**
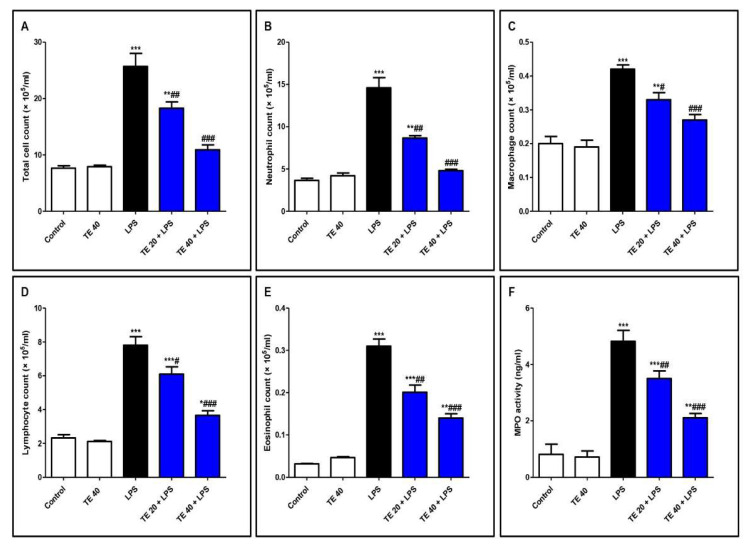
Terretonin (TE) ameliorated lipopolysaccharide (LPS)-induced inflammatory cell infiltration into the lung and suppressed myeloperoxidase (MPO) activity. (**A**–**E**) Total and differential cell counts in bronchoalveolar lavage fluid (BALF). (**F**) MPO activity in lung tissue. Data are the mean ± SE (*n* = 8). * *p* < 0.05, ** *p* < 0.01, *** *p* < 0.001 vs. control group; # *p* < 0.05, ## *p* < 0.01, ### *p* < 0.001 vs. LPS group (one-way ANOVA followed by Tukey’s Kramer multiple comparisons test).

As shown in Figure 4, LPS significantly intensified the inflammatory cells’ infiltration into the pulmonary tissue as there was an elevation in the differential and total cell counts in BALF compared to control mice. In addition, MPO activity was enhanced in the lungs of the LPS group. On the other hand, pretreatment with TE significantly reduced the total and differential cell counts, besides MPO activity compared to the LPS group.

### 3.3. TE Attenuated LPS-Produced Lipid Peroxidation and Augmented Antioxidants

**Figure 5:**
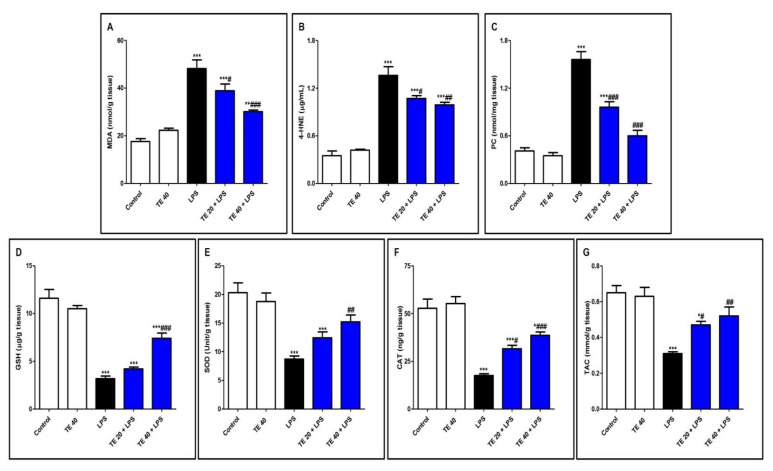
Terretonin (TE) attenuated lipopolysaccharide (LPS)-induced lipid peroxidation and augmented antioxidants in the lungs. (**A**) Malondialdehyde (MDA); (**B**) 4-hydroxynonenal (4-HNE); (**C**) protein carbonyl (PC); (**D**) reduced glutathione (GSH); (**E**) superoxide dismutase (SOD); (**F**) catalase (CAT); (**G**) total antioxidant capacity (TAC). Data are the mean ± SE (*n* = 8). * *p* < 0.05, ** *p* < 0.01, *** *p* < 0.001 vs. control group; # *p* < 0.05, ## *p* < 0.01, ### *p* < 0.001 vs. LPS group (one-way ANOVA followed by Tukey’s Kramer multiple comparisons test).

Injecting LPS significantly exaggerated the lipid peroxidative parameters, 4-HNE, MDA, and PC in the pulmonary tissue in contrast to the control group (Figure 5A–C). Furthermore, LPS depressed pulmonary antioxidants (GSH, SOD, CAT, and TAC) in comparison to the control group (Figure 5D–G). Notably, TE pretreatment produced a significant increase in the antioxidants, as well as a significant decrease in the lipid peroxidative parameters compared to the LPS group.

### 3.4. TE Counteracted LPS-Induced Suppression of SIRT1/Nrf2 Signaling and Targeted Genes in Lung

**Figure 6:**
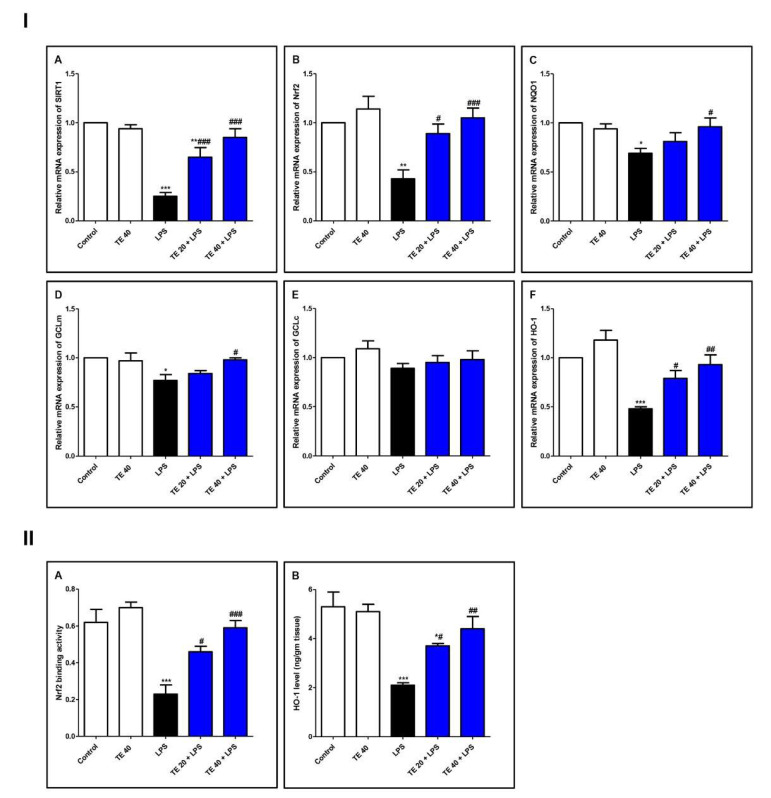
Terretonin (TE) counteracted the LPS-induced suppression of SIRT1/Nrf2 signaling and targeted genes in the lung. (**I**) (**A**–**F**) Gene expressions of SIRT1, Nrf2, NQO1, GCLm, GCLc, and HO-1. (**II**) (**A**) Nrf2 binding activity; (**B**) HO-1 level in the lung. Data are the mean ± SE (*n* = 8). * *p* < 0.05, ** *p* < 0.01, *** *p* < 0.001 vs. control group; # *p* < 0.05, ## *p* < 0.01, ### *p* < 0.001 vs. LPS group (one-way ANOVA followed by Tukey’s Kramer multiple comparisons test).

As demonstrated in Figure 6, LPS induced a remarkable reduction in the mRNA expressions of SIRT1, Nrf2, and its targeted genes (*GCLm, NQO1*, and *HO-1*) compared with the control group. Consequently, LPS significantly attenuated the Nrf2 binding activity and the level of HO-1 (Figure 6II). TE pretreatment significantly boosted the mRNA expression of SIRT1, GCLm, Nrf2, NQO1, and HO-1 in comparison to the LPS group. Additionally, TE augmented the Nrf2 binding activity and increased the HO-1 level, compared to the LPS group.

### 3.5. TE Suppressed LPS-Induced Activation of NF-κB/NLRP3 Signaling and Its Downstream Pro-Inflammatory Markers in Lung

**Figure 7:**
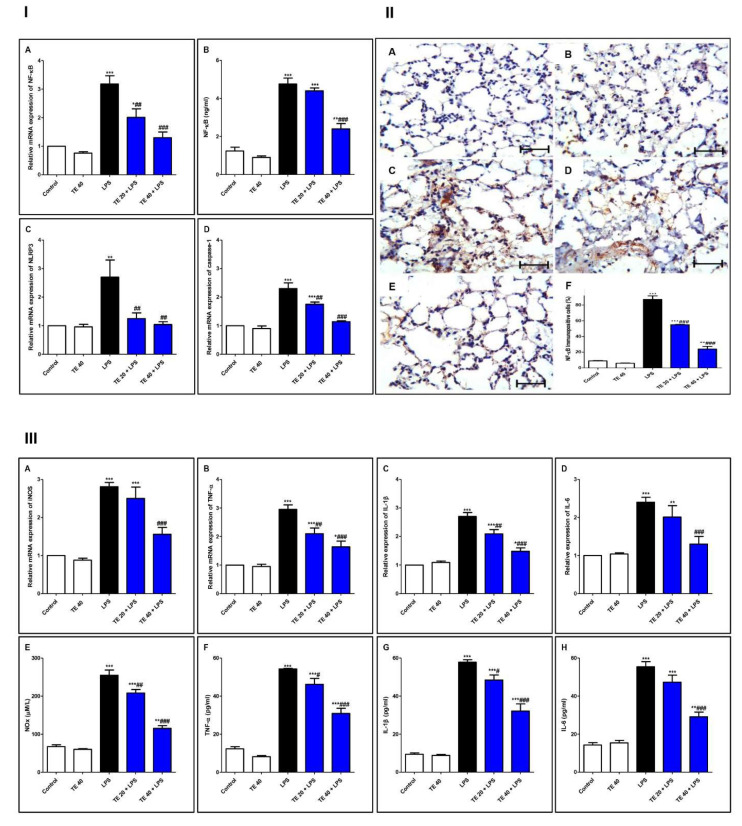
Terretonin (TE) suppressed the LPS-induced activation of NF-κB/NLRP3 signaling and its downstream pro-inflammatory markers in the lung. (**I**) (**A**) Gene expression; (**B**) level of NF-κB; (**C**) gene expression of NLRP3; (**D**) gene expression of caspase-1. (**II**) Immuno-expression of NF-κB protein: (**A**,**B**) lung sections of both the control and TE 40 groups, showing minimal cytoplasmic brownish staining; (**C**) LPS group showing marked nuclear and cytoplasmic brown immunostaining of inflamed lung tissue; (**D**) TE 20 + LPS and (**E**) TE 40 + LPS groups exhibiting marked improvement with moderate to minimal nuclear and cytoplasmic brown staining; NF-ĸB immunostain ×400, scale bar 25 µm; (**F**) % of immunopositive cells among different groups. (**III**) (**A**–**D**) Gene expression of iNOS, TNF-α, IL-1β, and IL-6; (**E**–**H**) levels of NOx, TNF-α, IL-1β, and IL-6. Data are the mean ± SE (*n* = 8). * *p* < 0.05, ** *p* < 0.01, *** *p* < 0.001 vs. control group; # *p* < 0.05, ## *p* < 0.01, ### *p* < 0.001 vs. LPS group (one-way ANOVA followed by Tukey’s Kramer multiple comparisons test).

As shown in Figure 7, injecting LPS remarkably enhanced the gene and protein expression of NF-κB, as well as the gene expression of NLRP3 and caspase-1 as compared to the control group. Additionally, LPS potentiated the gene expression of iNOS, as well as the gene expression of inflammatory mediators (IL-1β and -6 and TNF-α), compared to the control group. The levels of NOx, IL-6 and -1β, and TNF-α were remarkably elevated in the LPS group, in comparison to the control group.

Nevertheless, TE-pretreated groups revealed a remarkable inhibition of gene and immuno-expression of NF-κB, as well as its level. TE also inhibited LPS-caused activation of the NLRP3 inflammasome (NLRP3, caspase-1, and IL-1β). Furthermore, the gene expression and the level of inflammatory mediators were attenuated in TE-pretreated groups in comparison to the LPS group.

### 3.6. TE Decreased LPS-Induced Apoptosis

**Figure 8:**
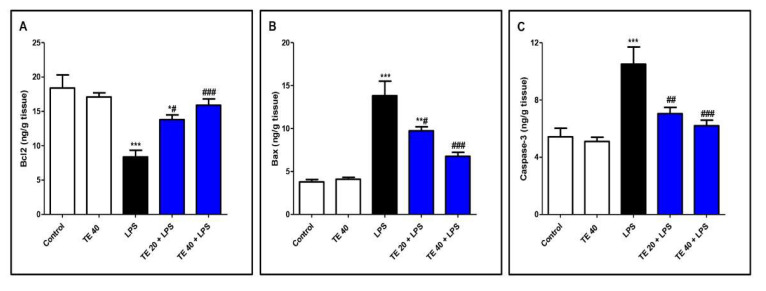
Terretonin (TE) decreased LPS-induced pulmonary apoptosis. (**A**) B-cell leukemia/lymphoma 2(Bcl-2); (**B**) Bcl-2-associated X protein (Bax); (**C**) caspase 3. Data are the mean ± SE (*n* = 8). * *p* < 0.05, ** *p* < 0.01, *** *p* < 0.001 vs. control group; # *p* < 0.05, ## *p* < 0.01, ### *p* < 0.001 vs. LPS group (one-way ANOVA followed by Tukey’s Kramer multiple comparisons test).

As shown in Figure 8, LPS administration decreased the level of the anti-apoptotic protein Bcl2 and increased the levels of the pro-apoptotic protein, Bax, as well as caspase-3 compared to normal mice. In contrast, TE pretreatment suppressed the apoptotic changes, as presented by a significant increase in Bcl2 and significant decreases in Bax and caspase-3 compared to the LPS group.

## 4. Discussion

Acute respiratory distress syndrome (ARDS), the clinical severe form of ALI, is a life-threatening condition, which has lacked innovative therapies until now. Alveolar-capillary barrier disturbance associated with the extended inflammatory reaction in the lung tissue results in an extreme gas exchange impairment. In the current study, the protective potential of TE toward LPS-induced ALI was investigated. The results demonstrated the ability of TE to attenuate LPS-caused lung injury via its potent antioxidant and anti-inflammatory properties.

Pulmonary edema occurs due to the activation and infiltration of neutrophils and macrophages, causing permeability of the capillary and alveolar barrier. This can be detected by the increase in the total protein content of BALF and lung W/D ratio due to the exudation of proteins from plasma [9,10,41]. Our results revealed that LPS induced pulmonary edema with an apparent aggravation in protein content of BALF and lung W/D ratio. Notably, an alleviation in pulmonary edema was observed in TE-treated groups. The damage in the cellular integrity of the pulmonary tissue was apparent through the elevated level of LDH in BALF of LPS-intoxicated animals. These findings are in coordination with the former investigations [1,8]. Notably, TE treatment suppressed the increase in LDH and hence preserved the cellular integrity of the lung.

Inflammatory mechanisms play a crucial role to clear pathogens from the body, but an excessive and persistent release may affect the tissues [4,42]. The damage occurs due to polymorphonuclear cells sequestration as they cause the deliverance of other mediators such as cytokines, chemokines, ROS/RNS, and Toll-like receptor involvement [43]. During ALI, the infiltration and accumulation of inflammatory cells specifically, neutrophils occur. Neutrophil emigration and activation are the earliest response to injury or inflammation, which lead to capillary permeability and edema. They amplify the inflammatory response by recruiting more leukocytes by the activation of chemokines [44]. Neutrophils contribute to chronic inflammation and immunological response by activating the liberation of pro-inflammatory mediators such as cytokines, chemokines, and metalloproteinases. Clinically, the severity of ALI is correlated with the inflammatory cells’ number in the BALF [45]. MPO is a good indicator for the estimation of neutrophils in lung tissue as it is a glycoprotein found in its intracellular granules. The activation of neutrophils releases MPO, so its accumulation reflects the damage to the lung tissue [46]. Our results showed a remarkable increase in the inflammatory cells in BALF after LPS administration, besides a marked increase in the MPO level. The damaging effect of LPS was further confirmed via histopathological examination, which revealed remarkable inflammatory lesions of the lung specimen. On the contrary, TE pretreatment resulted in the attenuation of the infiltration of inflammatory cells into the lung tissue, along with a depressed level of MPO. These biochemical results were further supported by the notable improvement of the histopathological picture of the lung tissue. These data demonstrated the potential protective effect of TE against LPS-produced ALI. To unravel the probable molecular mechanisms of this protective potential of TE, oxidative stress and inflammation modulators were the primary consideration to explore.

Oxidative stress plays a prominent function in the pathogenesis of LPS-induced ALI, as described by previous investigations [3,8,13,47]. ROS strike different organelles, causing lipid peroxidation, mutation in the DNA molecule, and inactivation of proteins. The accumulation of ROS potentiates the development of pulmonary edema and infiltration of inflammatory cells [48]. Neutrophil activation produces superoxide anions by undergoing an oxidative burst. Thus, the respiratory burst and release of ROS lead to many toxic pathophysiological consequences. The ROS lipid peroxidative effect will damage the parenchymal cells and the capillary basement membrane. Our results confirmed the elevation in the lipid peroxidative markers (MDA, 4-HNE, and PC) and reduced endogenous antioxidants (GSH, SOD, CAT, and TAC) in the lung tissues of the LPS group. These results agree with the findings of many previous studies [2,3,49,50]. TE pretreatment suppressed oxidative stress, and hence, the lipid peroxidative markers were ameliorated and the antioxidants were enhanced. This indicated the potential antioxidant activity of TE against LPS-induced oxidative stress.

SIRT1 plays a substantial function in the modulation of inflammatory injuries through potentiation of antioxidant defense mechanisms and blocking the release of pro-inflammatory cytokines. It was stated that SIRT1 regulates the signaling of the important antioxidant transcription factor Nrf2 [17]. SIRT1 also interferes with NF-κB signaling to negatively impact the downstream inflammatory cascade through blocking p65 nuclear translocation via deacetylation of NF-κB p65 [16,51]. Nrf2 is a powerful suppressor of oxidative/electrophilic stress and the inflammatory response. Under oxidative stressful conditions, the activation of Nrf2 boosts the expression of cytoprotective antioxidants genes such as *NQO1*, *GCL*, and *HO-1*, which is a remarkable stress protein involved in maintaining cellular homeostasis. However, under extreme conditions of oxidative stress, Nrf2 remains within the cytoplasm [52].

The impact of activation of the Nrf2/HO-1 pathway on the suppression of LPS-induced pulmonary inflammation has been documented previously in many studies [15,17,53,54]. In harmony with previous reports, our results indicated that LPS induced the downregulation of SIRT1/Nrf2/NQO1/GCL/HO-1 mRNA expression, as well as Nrf2 binding activity and HO-1 level. On the other hand, TE markedly enhanced the mRNA expression of SIRT1/Nrf2 and its targeted genes simultaneously with an elevated Nrf2 binding capacity and HO-1 level. These results suggested that the potent antioxidant activity of TE may be correlated to SIRT1/Nrf2 potentiation, and this may in part be responsible for the protective role of TE against LPS-induced injury.

LPS induces the activation of NF-ĸB by phosphorylation, leading to the upregulation of target inflammatory genes such as COX-2, iNOS, and cytokines such as TNF-α and ILs, as well as ROS production [55,56]. TNF-α is produced primarily after the stimulation of monocytes and macrophages by LPS that generate the inflammatory cascade by inducing cells to secrete cytokines and chemokines such as IL-6, causing the recruitment of more PMNs, further damaging the tissue [10,57,58]. Moreover, ROS act as a second messenger, controlling various downstream signaling mediators, including NF-ĸB, to augment the inflammatory responses [48,59]. LPS induces iNOS-acquired NO overproduction by alveolar inflammatory cells, resulting in alveolar-capillary barrier dysfunction and pulmonary edema in LPS-induced ALI. The concentration of NOx in BALF and the pulmonary iNOS expression from patients with acute respiratory distress syndrome are remarkably elevated than those in normal subjects [60]. Additionally, NF-ĸB mediates the activation of the NLRP3 inflammasome, which is the cornerstone of the inflammatory response. The NLRP3 inflammasome induces cytokine maturation and secretion. During ALI, the production of a mature active form of IL-1β is highly dependent on NLRP3 activation. IL-1β plays a vital role in ALI-associated edema via increased alveolar epithelial permeability [11,61]. Deletion of the NLRP3 inflammasome has a protective effect against ALI via the attenuation of lung epithelial cell death, inhibition of the recruitment of inflammatory cells, and proinflammatory cytokines production [62]. Furthermore, there is a correlation between the activation of the Nrf2/HO-1 signaling and the inhibition of the NLRP3 inflammasome activation, resulting in the reduction in IL-1β production and anti-inflammatory cytoprotective activities [63]. Our results align with the above studies that confirmed the augmentation of inflammatory NF-ĸB/NLRP3 signaling in the LPS-intoxicated group. The aggravation of NF-ĸB/NLRP3 activation and subsequent rise in the expressions of TNF-α, iNOS, and IL-6 and -1β were notable. Consequently, the levels of NOx and other cytokines were remarkably high in the lung tissue of the LPS group. Additionally, the pivotal role of NF-κB pathway stimulation in mediating the cellular apoptosis has been documented in the case of LPS-induced ALI [8]. NF-κB is vital for p53-mediated cell death, and RelA expression promotes apoptosis. Furthermore, the activation of NF-κB participates in the intrinsic pathway of apoptosis, although TNFα, an activator of NF-κB, is part of an extrinsic pathway of apoptosis [64]. The major players of the apoptosis process are Bcl2 proteins, which include pro-apoptotic proteins, such as Bax, and anti-apoptotic proteins such as Bcl-2 [65]. Caspases (a family of proteases), mainly caspase-3, contribute to the apoptotic process as they are responsible for the characteristic morphological and biochemical alterations of apoptosis [64,66,67]. Our results confirmed the elevation in apoptotic parameters in LPS-treated animals. Interestingly, a marked alleviation in the expression and level of these inflammatory and apoptotic mediators was observed in TE pretreated groups, suggesting that the modulation of NF-ĸB/NLRP3 signaling may contribute to the protective potential of TE.

The limitations of the present study include the use of TE as a pretreatment protective therapy against ALI. Further research is needed to evaluate the ability of TE to reverse the injurious effects of LPS.

## 5. Conclusions

**Figure 9:**
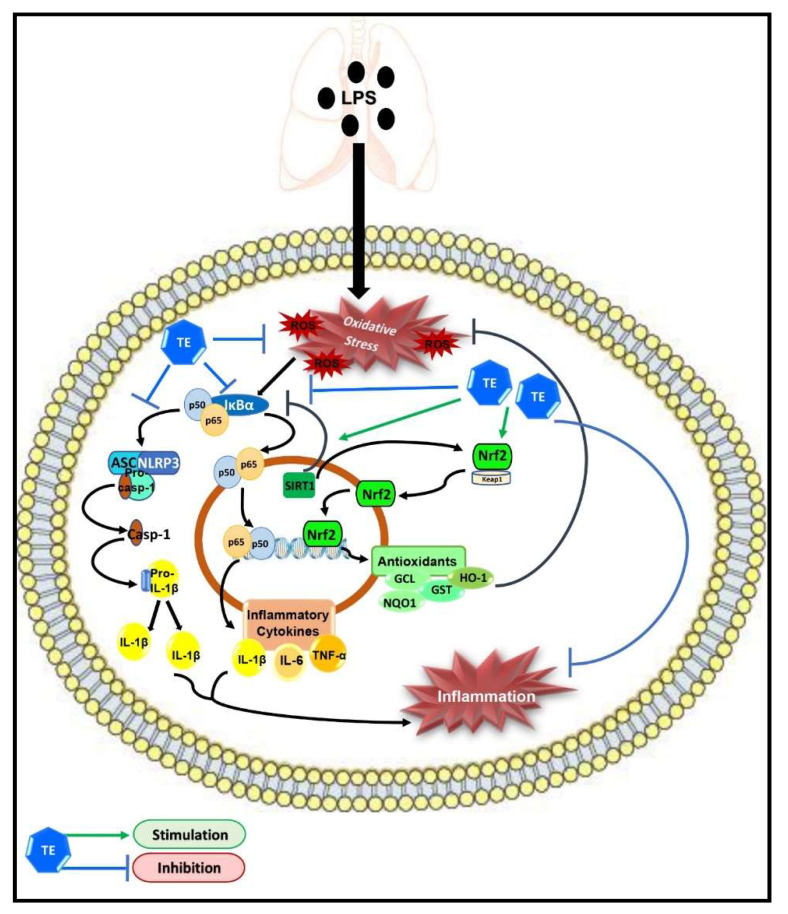
Schematic diagram demonstrating the molecular targets of terretonin (TE), which control its protective activity against LPS-induced acute lung injury.

Taken together, our results demonstrated the protective potential of TE against LPS-induced ALI, which may be due to its potent anti-inflammatory and antioxidant effects; therefore, it can be considered as a potential candidate for pulmonary inflammatory diseases. The underlying molecular targets of TE may still not be fully understood. However, our study demonstrated that TE can enhance SIRT1/Nrf2 signaling and its downstream antioxidant genes. Additionally, TE can inhibit the activation of NF-ĸB/NLRP3 inflammatory cascades (Figure 9). However, further investigations are needed to further elucidate other possible molecular mechanisms of TE.

## Figures and Tables

**Table 1:** Primers’ sequences used in RT-PCR measurements.

Gene (Mouse)	Accession No.	Sense Primer (5′–3′)	Antisense Primer (5′–3′)	PCR Product (bp)
*SIRT1*	NM_019812.3	CGATGACAGAACGTCACACG	ATTGTTCGAGGATCGGTGCC	111
*Nrf2*	NM_010902	AAGAATAAAGTCGCCGCCCA	AGATACAAGGTGCTGAGCCG	170
*NQO1*	NM_008706	CATTGCAGTGGTTTGGGGTG	TCTGGAAAGGACCGTTGTCG	111
*GCLm*	NM_008129	TAAGAAGGCGGCTTGATGCT	TGTGGTGAGTCCAACTGAGC	131
*GCLc*	NM_010295	CTTTGGGTCGCAAGTAGGAAGC	GGGCGTCCCGTCCGTTC	182
*HO-1*	NM_010442	CCTCACAGATGGCGTCACTT	TGGGGGCCAGTATTGCATTT	200
*NF-ĸB*	AY521463.1	AGGAAGGCAAAGCGAATCCA	TCAGAACCAAGAAGGACGCC	102
*iNOS*	NM_010927.4	GGTGAAGGGACTGAGCTGTTA	TGAAGAGAAACTTCCAGGGGC	163
*TNF-α*	NM_011609.4	GCTGTTGCCCCTGGTTATCT	ATGGAGTAGACTTCGGGCCT	102
*IL-1β*	NM_008361.4	GCCACCTTTTGACAGTGATGAG	AGCTTCTCCACAGCCACAAT	186
*IL-6*	NM_031168.2	AGTCCTTCCTACCCCAATTTCC	GGTCTTGGTCCTTAGCCACT	79
*NLRP3*	NM_145827.4	TGGGTTCTGGTCAGACACGAG	GGCGGGTAATCTTCCAAATGC	299
*Caspase-1*	NM_009807.2	GGACCCTCAAGTTTTGCCCT	GCAAGACGTGTACGAGTGGT	103
*β-actin*	NM_007393.5	TGAGCTGCGTTTTACACCCT	GCCTTCACCGTTCCAGTTTT	198

## Data Availability

Not applicable.
